# The economic and fiscal impact of incremental use of cell-based quadrivalent influenza vaccine for the prevention of seasonal influenza among healthcare workers in Italy

**DOI:** 10.1186/s12961-024-01122-w

**Published:** 2024-03-22

**Authors:** Giovanna Elisa Calabrò, Filippo Rumi, Roberto Ricciardi, Americo Cicchetti

**Affiliations:** 1 Section of Hygiene, Department of Life Sciences and Public Health, Università Cattolica del Sacro Cuore, L.Go F. Vito 1, 00168 Rome, Italy; 2 VIHTALI (Value in Health Technology and Academy for Leadership & Innovation), Spin-Off of Università Cattolica del Sacro Cuore, 00168 Rome, Italy; 3 Graduate School of Health Economics and Management (ALTEMS), Università Cattolica del Sacro Cuore, 00168 Rome, Italy

**Keywords:** Economic impact, Influenza vaccination, Healthcare workers, Cell-based quadrivalent influenza vaccine

## Abstract

**Background:**

Seasonal influenza has a significant impact on public health, generating substantial direct healthcare costs, production losses and fiscal effects. Understanding these consequences is crucial to effective decision-making and the development of preventive strategies. This study aimed to evaluate the economic and the fiscal impact of implementing an incremental strategy for seasonal influenza prevention using the cell-based quadrivalent influenza vaccine (QIVc) among healthcare workers (HCWs) in Italy.

**Methods:**

To estimate the economic impact of implementing this strategy, we performed a cost analysis that considered direct healthcare costs, productivity losses and fiscal impact. The analysis considered a 3-year time horizon. A deterministic sensitivity analysis was also conducted.

**Results:**

Assuming a vaccination coverage rate of 30% among HCWs, the analysis considered a total of 203 018 vaccinated subjects. On analysing the overall differential impact (including direct costs, indirect costs and fiscal impact), implementing QIVc vaccination as a preventative measure against influenza among HCWs in Italy would yield societal resource savings of €23 638.78 in the first year, €47 277.56 in the second year, and €70 916.35 in the third year, resulting in total resource savings of €141 832.69.

**Conclusions:**

The study demonstrated that implementing the incremental use of QIVc as part of a preventive strategy for seasonal influenza among HCWs in Italy could yield positive economic outcomes, especially in terms of indirect costs and fiscal impact. The resources saved could be utilized to fund further public health interventions. Policy-makers should consider these findings when making decisions regarding influenza prevention strategies targeting HCWs.

## Introduction

Influenza, a seasonal respiratory illness characterized by high rates of morbidity and mortality, places a substantial burden on healthcare resources and economies globally [1]. The WHO estimates that influenza causes from 290 000 to 650 000 respiratory deaths worldwide every year [2]. In Europe, influenza results in 4–50 million symptomatic cases annually, causing ~ 15 000 to 70 000 deaths and 150 000 hospital admissions [3].

As healthcare workers (HCWs) are in frequent contact with patients, they are at high risk of acquiring influenza and of potentially spreading the virus to individuals who are more susceptible to severe illness [4]. Therefore, protecting HCWs through vaccination is crucial to minimizing the risk of influenza transmission, safeguarding vulnerable individuals within healthcare settings and reducing the absenteeism and presenteeism associated with influenza-like illness (ILI) among HCWs [5]. Although it is recommended that, to protect patients, the optimal influenza vaccination coverage rate among HCWs be around 90%, coverage rates among HCWs worldwide are estimated to vary between 2% and 44% [4]. Furthermore, despite the availability of specific vaccination programs for HCWs in several European countries, a significant proportion of HCWs remain unvaccinated and therefore susceptible to influenza [6, 7]. Indeed, according to a recent European report, compliance with seasonal influenza vaccination among HCWs during the 2015–2016, 2016–2017 and 2017–2018 seasons was insufficient. Vaccination rates varied across countries, ranging from 63.2% in the UK (England) to 15.6% in Italy (median 30.2%) [8]. However, it did not report the reasons behind this variability in vaccination coverage.

In Italy, influenza vaccine uptake increased considerably during the 2020–2021 influenza season, partly owing to the impact of the severe acute respiratory syndrome coronavirus 2 (SARS-CoV-2) pandemic. Unfortunately, however, vaccination rates subsequently decreased during the 2021–2022 influenza season [9]. Hence, it is crucial to investigate the factors that influence the acceptance of vaccinations among HCWs and to evaluate the impact of the coronavirus disease 2019 (COVID-19) pandemic on influenza vaccination coverage in this specific group [9]. Sustaining a positive influenza vaccination trend among HCWs is essential, as they are role models for health behaviour and their actions serve as examples for patients. Moreover, HCWs have a responsibility to protect themselves to safeguard their vulnerable patients [10].

Vaccination does not only protect the individual against influenza; it also indirectly protects communities and contributes to the sustainability of healthcare systems by reducing hospitalizations, medical costs and potential complications associated with the disease. Furthermore, influenza vaccination promotes health equity and benefits national economies by minimizing productivity loss due to work absenteeism and preserving overall wellbeing [10]. Therefore, when conducting economic evaluations, it is essential to take into account these extensive benefits. Thus, economic models can guide policy-makers in evaluating vaccination strategies on the basis of their value, including their overall benefits [11, 12].

Moreover, it is important for decision-makers to adopt a comprehensive societal perspective when evaluating the economic value of vaccines; this includes considering the fiscal impact of infectious diseases [13]. The fiscal health model assumes that enhanced productivity among HCWs leads to higher individual income, thereby generating additional tax revenues that can be reinvested in healthcare services and the workforce. Conversely, if an illness reduces individual productivity, it has detrimental effects not only on the production systems involved, but also on the national healthcare system [13].

Recently, we estimated the economic and fiscal impact of an influenza vaccination program for HCWs in Italy [14]. Our analysis, which assumed an initial vaccination coverage rate of 30% and an influenza attack rate of 4.4%, considered a total of 23 213 influenza cases among the Italian HCWs. On estimating a yearly increase of 10% in HCWs vaccination coverage over a period of 5 years, we concluded that substantial savings could be achieved. These included a decrease in productivity losses amounting to €4 475 497.16 and an increase in tax revenues of €327 158.84. These additional revenues could be utilized to finance other healthcare interventions [14].

However, our model did not incorporate the direct costs of procuring and administering influenza vaccines. Indeed, it may be crucial to assess the fiscal implications of influenza vaccination strategies according to the specific vaccines used, as the effectiveness of these vaccines can significantly influence the results of the model.

During the 2023–2024 influenza season, the Italian Ministry of Health recommended influenza vaccination for adults aged 18–59 years, including HCWs, with the following vaccines: standard-dose egg-based quadrivalent influenza vaccines (QIVe), quadrivalent recombinant influenza vaccine (QIVr) and cell-based quadrivalent influenza vaccine (QIVc) [15]. Furthermore, two Italian Health Technology Assessment (HTA) reports on QIVc have been published in recent years. The initial report was released in 2019 [16, 17], followed by the second report in 2023 [18].

In both HTA reports [16, 18], the authors conducted a comprehensive analysis and systematization of all available scientific evidence on the QIVc. The most recent version from 2023 [18] highlighted that QIVc is immunogenic, effective and safe for individuals aged 2–64 years. Additionally, the authors concluded that the introduction of QIVc in Italy for individuals within the 2–64 year age range was highly cost-effective from both the perspectives of the National Health Service (NHS) and society. Ethically, the benefits in terms of health improvement, enhanced quality of life and reduced morbidity/mortality levels supported the recommendation for utilizing this health technology from the age of 2 years up to 64 years. This reassessment underscored how QIVc can serve as an evidence-based and value-driven choice for influenza vaccination across the Italian paediatric, adolescent and adult populations [18].

HTA stands as a crucial evidence-based method for assessing new vaccines. Demonstrating the value of newly developed vaccines is essential to informing decisions regarding their introduction and implementation in the healthcare setting, ensuring their proper utilization [17]. This is particularly pertinent in the case of influenza vaccination, as different vaccines are available, and various target groups necessitate immunization. Existing HTA reports on influenza vaccines can aid decision-makers in crafting recommendations for seasonal influenza vaccination. Similarly, the implementation of influenza vaccines in specific target groups, such as HCWs, requires updated evidence, including pharmacoeconomic considerations.

Therefore, the main objective of the present study was to estimate the economic and fiscal impact of implementing an incremental vaccination strategy using the QIVc for the prevention of seasonal influenza among HCWs in Italy.

## Methods

To assess the economic implications of implementing QIVc among HCWs in Italy, as opposed to QIVe, we conducted a budget impact analysis. The study adopted the following three-fold perspective:

1. Costs directly chargeable to the NHS;
2. Indirect costs, expressed as productivity losses due to work days lost on account of influenza;
3. Fiscal impact, expressed as lost tax revenues due to work days lost on account of influenza.

**Fig. 1:**
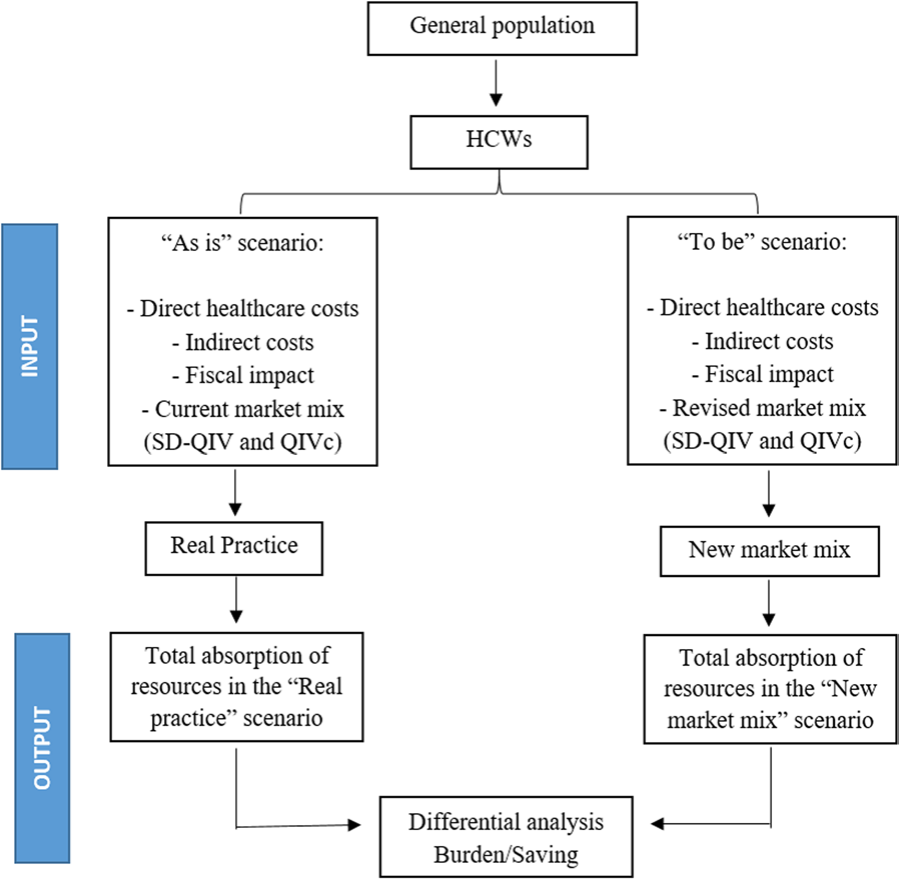
Model structure

Regarding the third perspective, we considered the fiscal impact framework proposed by Ruggeri et al. [13], which was also reported in our previous study [14]. Our analysis considered a 3-year time horizon. Figure 1 shows the structure of the model.

Specifically, the analysis involved a comparison between two alternative scenarios:

- Scenario ‘As is’, which assumed a constant market share of QIVc over the period considered;
- Scenario ‘To be’, which envisioned an incremental market share of QIVc over the period considered.

The impact on the budget was presented as a differential analysis of the two scenarios, taking into account direct costs, indirect costs and the fiscal impact resulting from the deployment of the vaccination strategy under study. Furthermore, to assess the robustness of the model results, a deterministic sensitivity analysis was carried out to determine the drivers whose variation most affected the estimates made in the base-case scenario. The parameters used in the model are described in the following paragraphs.

### Eligible population

We extrapolated the eligible population from the latest available 2021 data provided by the Italian National Institute of Statistics [19]. Table 1 reports the types of HCWs, their numbers per 10 000 inhabitants and their average annual salary. To estimate the annual salary of the HCWs, we referred to the Agency for the Negotiation Representation of Public Administrations, which reports the average salaries of different professionals in the health sector [20]. The total number of HCWs in Italy in 2021 was 676 727. As done in our previous model [14], we assumed a vaccination coverage rate of 30%, considering a total of 203 018 vaccinated HCWs.

**Table 1:** Eligible population considered in the model (latest available data referring to the Italian population in 2021 [19])

HCW types	HCWs per 10 000 Inhabitants	HCWs by type	Average annual salary
Anaesthetists	2.17	12 819	€84 037.00
Cardiologists	2.42	14 319	€84 037.00
Surgeons	1.41	8330	€84 037.00
Gastroenterologists	0.6	3529	€84 037.00
Geriatricians	0.74	4393	€84 037.00
Neurologists	1.17	6931	€84 037.00
Oncologists	0.81	4793	€84 037.00
Orthopaedists	1.6	9476	€84 037.00
Otorhinolaryngologists	0.77	4549	€84 037.00
Urologists	0.71	4174	€84 037.00
Other medical specialists	19.27	113 807	€84 037.00
Paediatricians	2.73	16 171	€84 037.00
General practitioners (GPs)	8.22	48 579	€84 037.00
Dentists	8.41	49 721	€84 037.00
Obstetricians	2.86	16 907	€72 512.00
Nurses	62.13	367 378	€33 733.00
Pharmacists	12.84	7791	€33 733.00
Total HCWs	127.29	676 727	€71 121.00

### Data input

With regard to direct costs, the model considered the costs of vaccine administration [21], the acquisition costs of the intervention (QIVc) and the comparator (QIVe), and the costs of influenza drugs [22, 23]. Regarding the fiscal impact, we first used the average salary of HCWs [19] to calculate productivity losses due to lost work days as a direct result of influenza, and then estimated the potential fiscal impact of absenteeism. In addition, the model considered an influenza attack rate of 4.4% in an unvaccinated cohort [24], a vaccination coverage rate of 30% in the HCWs group (assumption) and the vaccine effectiveness data necessary to identify the number of influenza cases among HCWs in each scenario (vaccination with QIVc or QIVe). To estimate the effectiveness of the vaccines, we referred to two main studies [25, 26]. We extrapolated QIVe effectiveness from Cai et al. [25] and weighted the effectiveness in relation to the proportions of influenza virus strains (96% type A, 4% type B [27]). Then, we derived QIVc effectiveness from the Puig-Barbera meta-analysis [26], which shows that QIVc is 11% more effective in preventing influenza outcomes. Indirect costs and fiscal impact were taken from our previous economic model, published in 2022 [14]. Specifically, assuming an average of 48 weeks of work and a total of 44 h per week, the model divided the hourly cost of each HCW into a fixed part on the basis of the gross taxable amount (83%) and a variable part (17%). These hours were quantified in monetary terms by applying the average salary presented in Table 1. The cost of 1 h of an HCW's work in Italy was determined to be €36.50, with a fixed part of €30.30 and a variable part of €6.21 being considered in the simulation. On the basis of this information, the model calculated an annual average gross taxable income of €77 089.83. Considering that influenza symptoms typically last for 2 days (based on conservative estimates from the Italian National Institute of Health data [28]), the model calculated productivity losses, in terms of hours lost owing to symptomatic influenza, to be 16 working hours. Consequently, influenza could potentially reduce the total annual taxable income of an HCW to €76 990.55 (from the original €77 089.83). The model also estimated an average annual tax revenue of €26 318.63 per HCW, compared with €26 275.94 per HCW who contracted influenza. Therefore, the estimated tax impact of influenza episodes was €42.69, while the impact on indirect costs (in terms of productivity losses) amounted to €584.01. Table 2 presents all the parameters used to populate the model.

**Table 2:** Input data used in our model

Variable	Value	Source
Vaccine administration cost	€6.16	[21]
QIVe acquisition cost	€6.55	NHS transfer price
QIVc acquisition cost	€7.50	NHS transfer price
Costs of prescribed influenza drugs	€20.78	[22]
Costs of influenza drugs without prescription	€11.34	[23]
Cost of GP visit	€20.66	Italian national tariff
Vaccination coverage among HCWs	30%	Assumption
Influenza attack rate in an unvaccinated cohort	4.4%	[24]
Number of working days lost (average duration of influenza)	2	Italian National Institute of Health, 2023 [28]
HCWs who develop influenza symptoms	66.90%	[25]
Vaccine effectiveness (QIVe)	45.91%	[25]
Vaccine effectiveness (QIVc)	56.91%	[25, 26]
Indirect costs and fiscal impact		
Total working hours/HCW	2112	[20]
Total working hours/week	44	[14]
Hourly cost	€36.50	Calculated
Taxable hourly fixed part	€30.30	Calculated
Taxable hourly variable part	€6.21	Calculated
Total weekly taxable income	€1606.04	Calculated
Total annual taxable income	€77 089.83	Calculated
Number of working hours lost owing to influenza	16	[14]
Working hours considering one episode of influenza/HCW	2096	[14]
Impact of influenza complications on total potential man-hours	0	[14]
% HCWs with influenza complications	0%	Assumption
Total annual taxable income of a HCW who contracts influenza	€76 990.55	Calculated
Personal income tax rates (Italy)		
€15 000	23.00%	[29]
€28 000	27.00%	[29]
€55 000	38.00%	[29]
€75 000	41.00%	[29]
> €75 000	43.00%	[29]
Annual revenue (no influenza)	€26 318.63	Calculated
Annual revenue (with influenza)	€26 275.94	Calculated
Tax impact	€42.69	Calculated
Annual cost of productivity loss per HCW	€584.01	Calculated

### Market share

As previously stated, we conducted a differential analysis between two distinct scenarios. The first scenario (‘As is’) assumed a constant market composition over the 3-year time horizon considered. In contrast, the second scenario (‘To be’) considered an incremental share of QIVc, compared with QIVe, over the same 3-year period. The market shares used in the base case are assumptions of the authors and are presented in Table 3.

**Table 3:** Market shares used in the model

	Year 1 (%)	Year 2 (%)	Year 3 (%)
Market share current market mix (‘As is’ scenario)			
QIVc	10	10	10
QIVe	90	90	90
Total	100	100	100
Market share revised market mix (‘To be’ scenario)			
QIVc	20	30	40
QIVe	80	70	60
Total	100	100	100

## Results

**Fig. 2:**
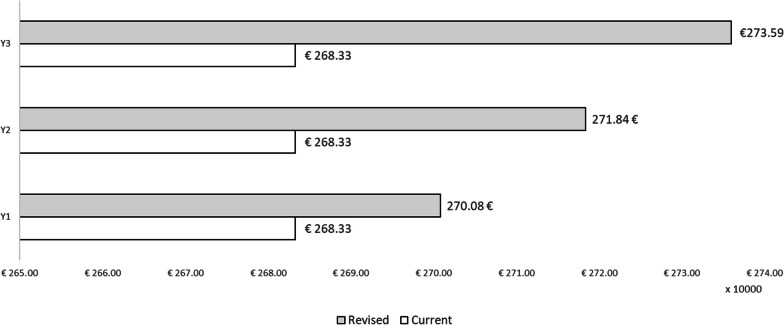
Direct healthcare costs

The results are reported as total direct costs, cases of influenza with symptoms among vaccinated HCWs, costs related to the resolution of influenza episodes, fiscal impact and indirect costs for each year of the period and for each of the two scenarios hypothesized. The result of the budget impact model is expressed as the difference between the scenario in which the share of QIVc administered to HCWs in Italy increases, and the scenario in which QIVe and QIVc maintain a constant share of the market. Table 4 presents the results of the ‘As is’ scenario and Table 5 those of the ‘To be’ scenario. With regard to direct healthcare costs, the slight increase in resource absorption due to the higher acquisition cost of QIVc than of QIVe has been highlighted (Fig. 2).

**Table 4:** Results of the current market mix (‘As is’ scenario)

Current market mix	Year 1	Year 2	Year 3
Population	203 018	203 018	203 018
Direct healthcare costs			
QIVe acquisition costs	€1 196 791.70	€1 196 791.70	€1 196 791.70
QIVc acquisition costs	€152 263.58	€152 263.58	€152 263.58
Vaccine administration costs	€1 250 591.50	€1 250 591.50	€1 250 591.50
Total acquisition and administration costs	€2 599 646.77	€2 599 646.77	€2 599 646.77
Influenza cases among vaccinated HCWs			
Vaccination with QIVe	4348	4348	4348
Vaccination with QIVc	385	385	385
Influenza cases with symptoms among vaccinated HCWs			
HCWs with influenza and symptoms	3167	3167	3167
Costs of prescribed influenza drugs	€44 021.45	€44 021.45	€44 021.45
Costs of influenza drugs without prescription	€24 023.26	€24 023.26	€24 023.26
Influenza-related GP visits^a^	€15 576.76	€15 576.76	€15 576.76
Total direct healthcare costs	€2 683 268.24	€2 683 268.24	€2 683 268.24
Fiscal impact			
QIVe	€124 193.12	€124 193.12	€124 193.12
QIVc	€10 993.36	€10 993.36	€10 993.36
Total fiscal impact	€135 186.48	€135 186.48	€135 186.48
Indirect costs (productivity losses)			
QIVe	€1 698 948.29	€1 698 948.29	€1 698 948.29
QIVc	€150 388.01	€150 388.01	€150 388.01
Total indirect costs	€1 849 336.31	€1 849 336.31	€1 849 336.31
Total (direct costs + indirect costs + fiscal impact)	€4 667 791.03	€4 667 791.03	€4 667 791.03

^a^Assuming that only 35% of HCWs who develop influenza symptoms visit a GP

**Table 5:** Results of the revised market mix (“To be” scenario)

Revised market mix	Year 1	Year 2	Year 3
Population	203 018	203 018	203 018
Direct healthcare costs			
QIVe acquisition costs	€1 063 814.84	€930 837.99	€797 861.13
QIVc acquisition costs	€304 527.15	€456 790.73	€609 054.30
Vaccine administration costs	€1 250 591.50	€1 250 591.50	€1 250 591.50
Total acquisition and administration costs	€2 618 933.49	€2 638 220.21	€2 657 506.93
Influenza cases among vaccinated HCWs			
Vaccination with QIVe	3865	3382	2899
Vaccination with QIVc	770	1155	1540
Influenza cases with symptoms among vaccinated HCWs			
HCWs with influenza and symptoms	3101	3035	2969
Costs of prescribed influenza drugs	€43 107.76	€42 194.07	€41 280.38
Costs of influenza drugs without prescription	€23 524.64	€23 026.02	€22 527.41
Influenza-related GP visits^a^	€15 253.46	€14 930.15	€14 606.85
Total direct healthcare costs	€2 700 819.35	€2 718 370.46	€2 735 921.57
Fiscal impact			
QIVe	€110 393.88	€96 594.65	€82 795.41
QIVc	€21 986.73	€32 980.09	€43 973.46
Total fiscal impact	€132 380.61	€129 574.74	€126 768.87
Indirect costs (productivity losses)			
QIVe	€1 510 176.26	€1 321 404.23	€1 132 632.19
QIVc	€300 776.03	€451 164.04	€601 552.05
Total indirect costs	€1 810 952.29	€1 772 568.27	€1 734 184.25
Total (direct costs + indirect costs + fiscal impact)	€4 644 152.25	€4 620 513.47	€4 596 874.69

^a^Assuming that only 35% of HCWs who develop influenza symptoms visit a GP

**Fig. 3:**
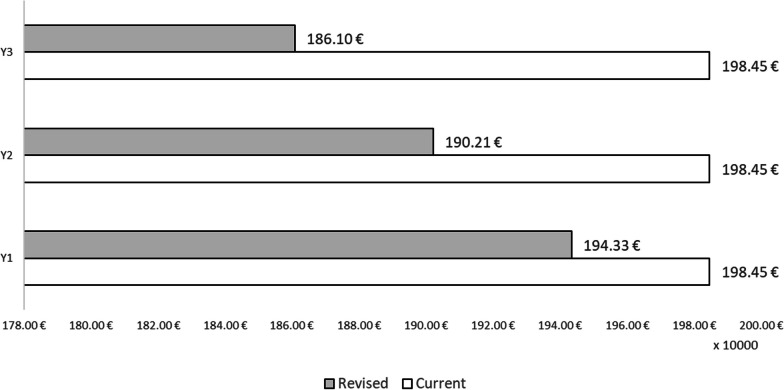
Indirect costs and fiscal impact

It should be noted that the model assumed that only 35% of HCWs who contract influenza develop symptoms. Therefore, the costs of influenza drugs and GP visits are only quantified for those who develop influenza symptoms. On broadening the perspective to indirect costs and fiscal impact, the higher direct costs incurred through the adoption of the ‘To be’ scenario are largely offset by incremental savings over the time horizon considered in the societal perspective (Fig. 3). Indeed, with regard to both fiscal impact and indirect costs, savings are incremental and reach their peak in the third year of the analysis.

**Fig. 4:**
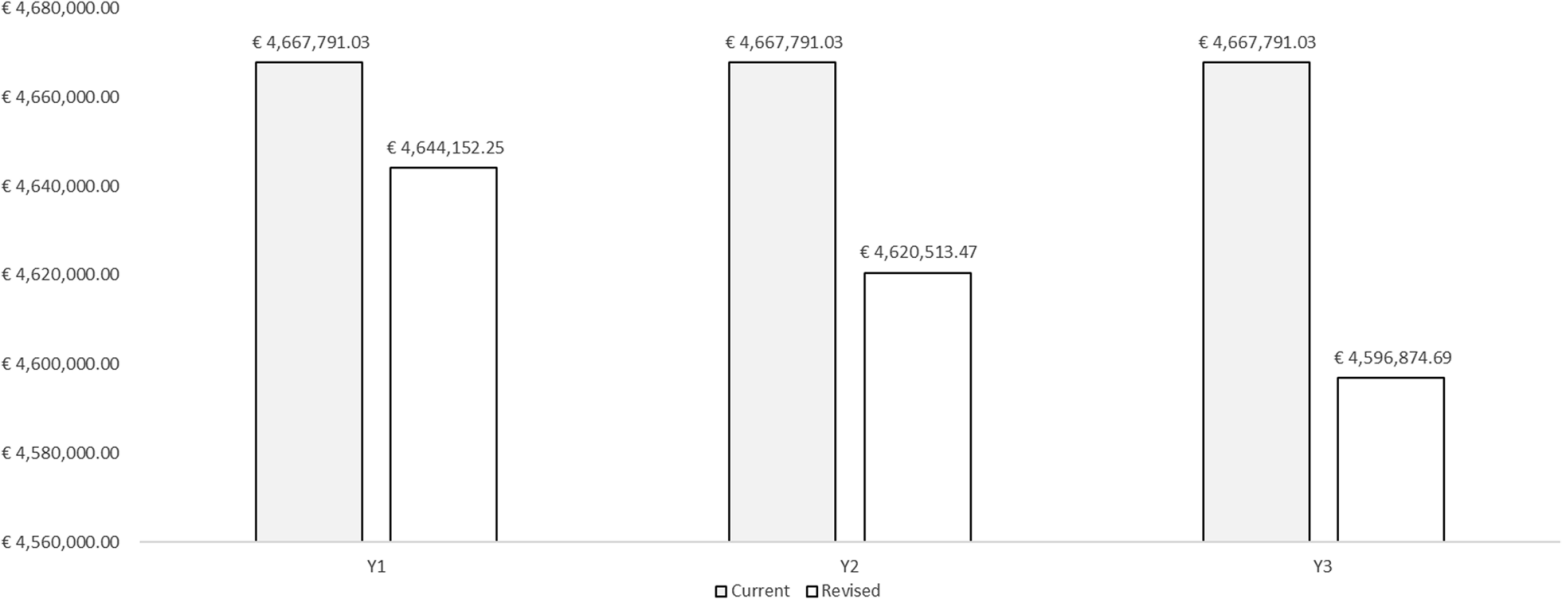
Total costs of the two scenarios considered in the model

**Fig. 5:**
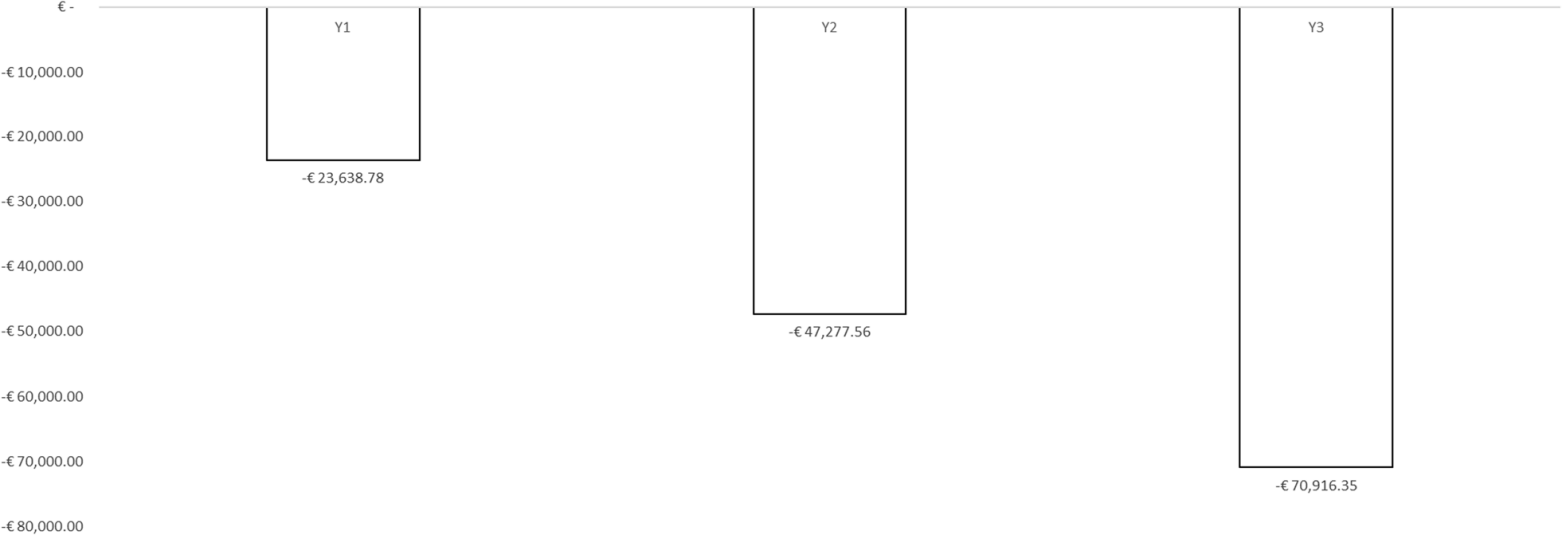
Budget impact results

Specifically, direct costs increase by €17 551.11 in the first year, €35 102.22 in the second year and €52 653.33 in the third year. By contrast, in terms of indirect costs and fiscal impact, there is an incremental savings of €41 189.89 in the first year, €82 379.78 in the second year and €123 569.67 in the third year. In global terms (direct costs, indirect costs and fiscal impact), the differential analysis shows that introducing QIVc as a preventive anti-influenza strategy among HCWs in the Italian setting would result in resource savings for society of €23 638.78 in the first year, €47 277.56 in the second year and €70 916.35 in the third year, which means total resource savings of €141 832.69. Figures 4 and 5 show the total costs and overall impact of the two scenarios considered.

### Deterministic sensitivity analysis

**Fig. 6:**
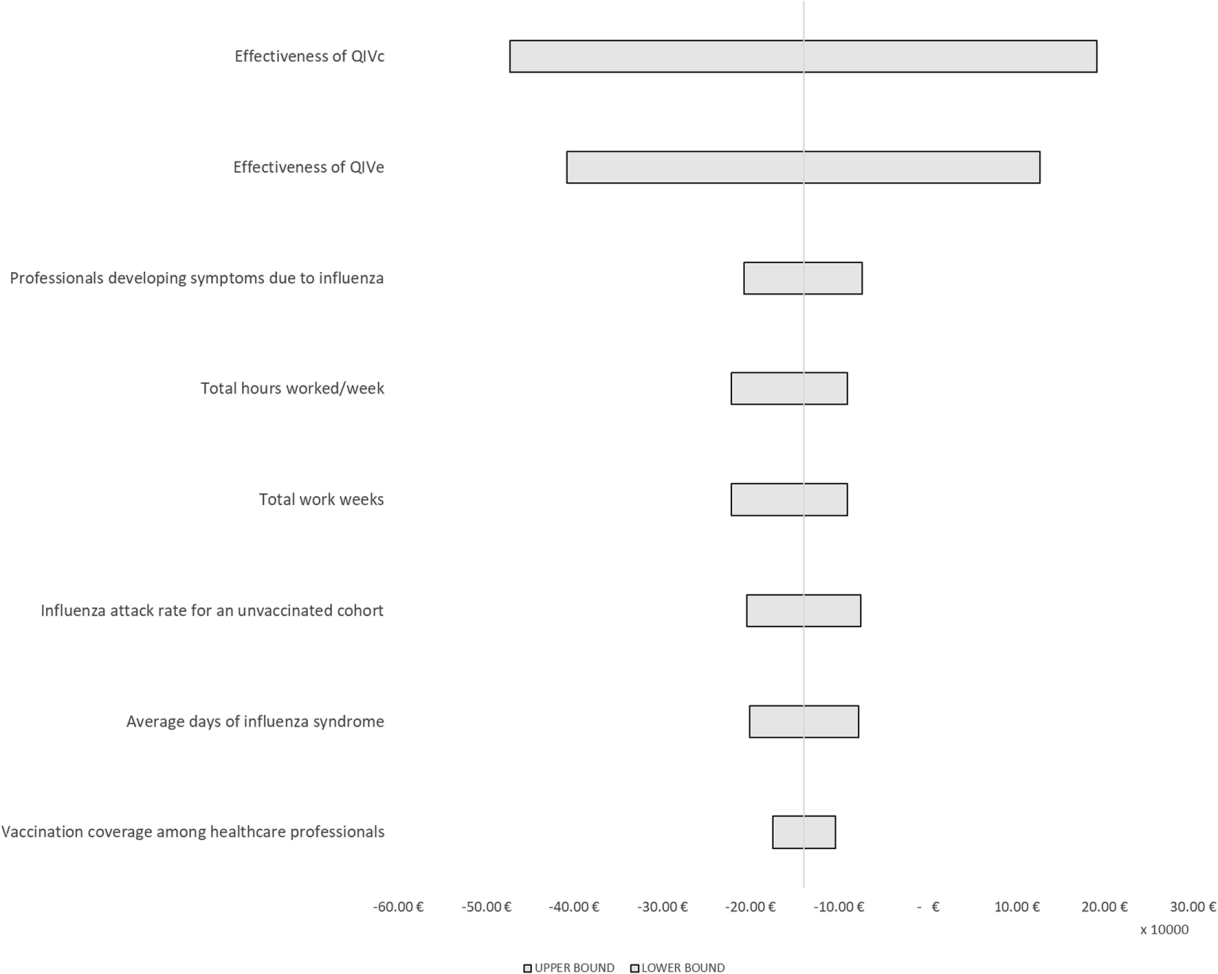
Deterministic sensitivity analysis

To characterize the uncertainty of the parameters used, we conducted a deterministic sensitivity analysis [30] in which we assumed a deviation of ±25% from the values entered into the model in the base case (Fig. 6). The parameters whose deviation would have the greatest impact on the results of the analysis are those related to the effectiveness of the vaccines considered (QIVc and QIVe) in preventing influenza. The results do not seem to be particularly sensitive to deviations in the parameters regarding HCWs who develop symptoms due to influenza, the influenza attack rate in an unvaccinated cohort, the average duration of influenza syndrome or the vaccination coverage rate among HCWs.

## Discussion

We estimated the economic impact of implementing an incremental influenza vaccination strategy with QIVc among Italian HCWs on considering direct healthcare costs, productivity losses and fiscal impact. The analysis considered a 3-year period and involved a comparison between two alternative scenarios: the ‘As is’ scenario, which assumed a constant market share of QIVc over the period considered, and the ‘To be’ scenario, which envisioned an incremental market share of QIVc over the 3 years considered. Assuming a vaccination coverage rate of 30%, we considered a total of 203 018 vaccinated HCWs.

Regarding direct healthcare costs, our analysis revealed a slight rise in resource absorption, owing to the higher acquisition cost of QIVc than of QIVe. However, these higher direct costs were largely offset by incremental savings over a 3-year time horizon from a societal perspective. Specifically, in terms of both fiscal impact and indirect costs, on increasing the share of QIVc, these savings gradually rose, reaching their peak in the third year of the period. Analysis of the overall differential impact (including direct costs, indirect costs and fiscal impact) revealed that implementing QIVc vaccination among HCWs in Italy would generate societal resource savings of €23 638.78 in the first year, €47 277.56 in the second year and €70 916.35 in the third year, resulting in total resource savings of €141 832.69. These resources could be utilized to fund interventions within the public health domain, thereby yielding additional improvements in healthcare. Consequently, this strategy could reduce productivity losses in the population, and in turn, increase tax revenues. Hence, an increase in vaccination coverage among HCWs not only results in increased tax revenues due to the lower number of professionals affected by influenza, but also reduces indirect costs (in terms of productivity losses) by minimizing the average number of workdays lost by HCWs.

Moreover, our results highlight the significance of investing in and utilizing influenza vaccines that are more effective. Indeed, the realization of a more substantial impact of vaccination in economic savings for both the health system and society is achievable only through the utilization of more effective vaccines. Given the obtained results, it is evident that prioritizing the promotion of influenza vaccination strategies among HCWs is imperative. This prioritization is essential for increasing vaccination coverage within this target population, ensuring health benefits for both healthcare professionals and their patients. Additionally, it contributes to the sustainability of health systems through a value allocation of health resources.

Therefore, to effectively highlight and document the broader value of vaccines, economic evaluations should be conducted from both the societal and the healthcare system perspectives [31]. Future economic assessments should place greater emphasis on evaluating the impact of vaccination on preventing complications and generating health benefits for HCWs and the broader advantages that vaccination offers to the community beyond individual protection [10]. These principles are perfectly in line with the framework of Broader Value of Vaccines proposed by Bell et al. in 2022 [11]. This framework classifies the effects of vaccines into four categories: (1) narrow health effects, which pertain to the impact of vaccines on the health of vaccinated individuals; (2) broad health effects, which concern the impact on the health of the unvaccinated population; (3) health system economic effects, which encompass the costs associated with vaccination and the corresponding budgetary offsets within the healthcare system; and (4) societal economic effects, which regard the broader economic impact of vaccines beyond the health system, such as effects on productivity and macroeconomic growth.

These different effects should also be considered in the processes of HTA [32]. Indeed, to improve the HTA of vaccines, several additional factors need to be considered, namely: the broader cost offsets within the healthcare system, the impact of vaccination on the quality of life of carers, its efficacy in curbing viral transmission, the prevention of antimicrobial resistance (AMR) and the macroeconomic effects of vaccination [11, 12]. Furthermore, there is a need for new economic models that can capture not only the mere cost–benefit ratio of vaccination, but also its broader value as an investment in health [33].

All stakeholders should possess a collective understanding of value in healthcare to maximize social wellbeing. In recent years, the focus has shifted from value-based healthcare to a value-based health system, and it has been recognized that the entire healthcare system plays a role in enhancing societal wellbeing through prevention and health promotion efforts [34, 35]. However, despite the proven effectiveness and cost-effectiveness of preventive interventions, many countries continue to allocate insufficient resources to prevention [36].

In our previous analysis [14], we demonstrated that enhancing influenza vaccination coverage among HCWs could reduce productivity losses and generate higher fiscal revenues. These revenues can be used to finance various health interventions, including the implementation of vaccination strategies among HCWs [14].

Health systems can effectively combat annual influenza epidemics, as numerous safe and effective vaccines are now available. However, to ensure appropriateness and sustainability, every individual should receive the appropriate vaccine [37]. The selection of a vaccine should also align with the appropriateness principle, which means utilizing the available products on the basis of the characteristics and health needs of different age groups and specific population groups [17, 37]. Indeed, the immunogenicity, efficacy and effectiveness of influenza vaccines are known to depend not only on the characteristics of the vaccine, but also on those of the host and the virus [38].

Among the available vaccines that have the potential to alleviate the substantial burden of influenza, QIVc presents additional opportunities [16–18]. In Italy, a recently published HTA report on QIVc [18] pointed out that much real-world evidence suggests that QIVc has advantages over QIVe in the population aged < 65 years. Specifically, in comparison with QIVe, the use of QIVc is associated with a significant reduction (25–29%) in cases of influenza due to the subtype A(H3N2). Furthermore, in subjects aged < 65 years, QIVc has, on average, proved more effective than QIVe in preventing hospitalizations and/or medical visits for clinically diagnosed influenza, pneumonia and other less specific outcomes [18]. In the same HTA report on QIVc, an economic evaluation was also conducted by implementing a dynamic transmission model; this revealed that the introduction of QIVc in Italy (in subjects aged 2–64 years) would be highly cost-effective or cost-saving (dominant) in both perspectives adopted in the analysis (NHS and society) and in every age group considered. The report concluded that the introduction of QIVc in Italy in subjects aged 2–64 years is ‘good value for money’ [18].

In this context, our economic model provided further evidence of the value of a vaccination strategy for HCWs on the basis of the use of QIVc. Obviously, the higher the vaccination coverage among HCWs by using a more effective influenza vaccine, the more resources can be saved and reinvested in other health interventions [14]. This approach is fundamental to the sustainability of the health systems and to the value-based allocation of health resources [35, 39]. Furthermore, according to this perspective, it is essential for health systems to consider all stakeholders: citizens and patients should have timely and equal access to more effective health technologies; it is crucial to encourage research and development that focuses on creating high-value technologies; decision-makers and policy-makers should endorse innovation by employing evidence-based tools for evaluation; and health systems must foster technological innovation while ensuring long-term sustainability [40].

In the light of our results, it is evident that prioritizing the implementation of influenza vaccination strategies among HCWs is crucial to enhancing vaccination rates within this specific group. This approach yields various advantages, including improved health protection for both HCWs and their patients and greater long-term viability of healthcare systems through the effective allocation of health resources [41]. In a similar vein, as highlighted by Boey et al. [42], there is a need for well-designed campaigns to combat seasonal influenza. These should emphasize the importance of educating individuals with regard to vaccination, effectively communicate the benefits of vaccination and facilitate access to vaccination services. Furthermore, it is crucial to recognize that these efforts should emphasize not solely the value for patients, but also the personal benefits for HCWs [42].

Our study has several limitations. First, a significant portion of our data were taken from the scientific literature or based on assumptions. However, to address the potential lack of robustness associated with the values used in the analysis, we conducted a deterministic sensitivity analysis. Moreover, it is noteworthy that our results are conservative. For instance, we only considered a loss of two working days due to influenza, while the literature suggests that the loss could be even greater (4–6 days according to some data [43]). Additionally, our analysis did not consider the economic impact of presenteeism, which is difficult to estimate. Furthermore, the study assumed an average salary of HCWs, without considering differences among different professional roles. To obtain more comprehensive information on indirect costs and the resulting fiscal impact, it would be advisable to employ a weighted average and to gather more specific data on those professionals who are most susceptible to influenza contagion.

A further limitation stems from the non-availability of effectiveness levels of the different vaccines in matched and mismatched years. Indeed, the parameters whose deviation would have the greatest impact on the results of our analysis are those regarding the effectiveness of the vaccines considered in the model. However, there is evidence that, overall, QIVc exhibits greater effectiveness than QIVe [18].

Lastly, another limitation is associated with the construction of a static model for our costs evaluation. Indeed, in the economic evaluation of influenza vaccines, prioritizing dynamic economic models over static models is crucial [44]. This preference stems from the tendency of static models to often underestimate the effectiveness and cost-effectiveness of immunization programs, particularly in terms of their indirect effects [44], as also explained in the guidance on the economic evaluation of influenza vaccination proposed by the WHO [45]. However, the dynamic model on QIVc had already been previously developed and published [18], and the objective of our new study was to evaluate, in particular, the fiscal impact of an immunization strategy for the Italian HCWs using the QIVc, all to highlight how traditional methods aimed at estimating the cost of illness from a social perspective can be improved by additionally considering the fiscal impact, which accounts for the decrease in fiscal revenues due to illness.

Despite these limitations, our economic model, which also explored the fiscal dimension, can enhance our understanding of the impact of influenza in the specific Italian context. Thus, it can help decision-makers to formulate vaccination policies that consider the comprehensive value of the various influenza vaccines available. It must be emphasized that acquiring new evidence on the value of different influenza vaccines is essential to promote their appropriate utilization and facilitate the implementation of evidence-based and value-based vaccination strategies, especially in risk categories such as HCWs.

## Conclusions

In high-risk groups such as HCWs, preventing influenza through vaccination offers us a unique opportunity to preserve people’s health and minimize the economic consequences of influenza on both healthcare systems and society. Specifically, vaccinating HCWs helps mitigate productivity losses and reduces the fiscal burden associated with the illness. Increased productivity leads to higher incomes, consumption and tax revenues, which can subsequently be channelled towards increased investments in healthcare. Promoting widespread influenza vaccination and implementing health policies aimed at improving vaccination coverage among HCWs should be priority actions in healthcare systems around the world, as well as ensuring their long-term sustainability.

Furthermore, our results underscore the importance of advocating for the adoption of immunization strategies utilizing the most effective influenza vaccines. This advocacy is essential for allocating healthcare resources in accordance with a value-based approach. In reality, achieving a more significant impact of vaccination in terms of economic savings for both the healthcare system and society is contingent upon the utilization of more effective vaccines.

Eventually, the development of vaccination programs based on the recognition of the broader value of influenza vaccines is essential. Attaining this goal necessitates the application of rigorous and evidence-based methodologies such as HTA. Additionally, the utilization and advancement of economic models capable of encompassing the full value of influenza vaccination play a fundamental role in this process.

Reinforcing the generation of evidence and data is crucial for shaping evidence-based vaccination policies. This relies on the adoption of new or enhanced assessment frameworks capable of acknowledging the broader value of influenza vaccines and vaccination. The transition towards a value-based vaccination approach should involve the active and informed participation of all pertinent stakeholders.

## Data Availability

The datasets during and/or analysed during the current study available from the corresponding author on reasonable request.
